# Associations between High Protein Intake, Linear Growth, and Stunting in Children and Adolescents: A Cross-Sectional Study

**DOI:** 10.3390/nu15224821

**Published:** 2023-11-17

**Authors:** Ting Xiong, Yuanjue Wu, Jiazhen Hu, Shiqi Xu, Yan Li, Binxuan Kong, Zhuangyu Zhang, Liangkai Chen, Yuhan Tang, Ping Yao, Jingfan Xiong, Yanyan Li

**Affiliations:** 1 Department of Nutrition and Food Hygiene, School of Public Health, Guangzhou Medical University, Guangzhou 511436, China; xiongkoala@gzhmu.edu.cn (T.X.); wyj@gzhmu.edu.cn (Y.W.); hjz15362662706@163.com (J.H.); wan109149@163.com (S.X.); 2 Shenzhen Center for Chronic Disease Control, Shenzhen 518020, China; nyan0727@outlook.com; 3 Department of Nutrition and Food Hygiene, Hubei Key Laboratory of Food Nutrition and Safety and the Ministry of Education (MOE) Key Laboratory of Environment and Health, School of Public Health, Tongji Medical College, Huazhong University of Science and Technology, Wuhan 430030, China; apricotkong@163.com (B.K.); 15703079104@163.com (Z.Z.); clk@hust.edu.cn (L.C.); 2015220157@hust.edu.cn (Y.T.); yaoping@mails.tjmu.edu.cn (P.Y.)

**Keywords:** high protein intake, height, linear growth, stunting, children and adolescents

## Abstract

Background and aims: Childhood and adolescence are critical periods for linear growth and preventing stunting. Current evidence indicates that dietary protein intake in children and adolescents is often two to three times higher than the recommendations in many regions worldwide. However, few studies have focused on the association between high protein intake and linear growth and stunting in this population. We aim to investigate this association in children and adolescents aged 6 to 18 years in a population with relatively high protein consumption. Methods: We conducted a large cross-sectional study involving 3299 participants from Shenzhen, a modern metropolis of China. Protein intake, including total protein, animal protein, and plant protein, was evaluated by a food-frequency questionnaire and expressed as grams per kilogram of body weight per day (g·kg^−1^·d^−1^) and as a percentage of total energy intake (%E). The primary outcomes were body height and height-for-age Z score (HAZ). Generalized linear models and logistic regression analyses were employed to examine the associations between protein intake and outcomes. We also conducted stratified analyses across different genders and pubertal stages in the aforementioned associations. Results: The mean protein intake was 1.81 g·kg^−1^·d^−1^ (17% E). After adjusting for serum calcium, zinc, vitamin D_3_, vitamin A levels, birth outcomes, lifestyle, and parental characteristics, each standard deviation increase of 1 in protein intake (0.64 kg^−1^·d^−1^) is found to be associated with a −5.78 cm change in body height (95% CI: −6.12, −5.45) and a −0.79 change in HAZ (95% CI: −0.84, −0.74). Consistent results were observed when protein intake was expressed as %E or specifically as animal or plant protein. Moreover, the relationship between protein intake and linear growth remained consistent across genders in different pubertal stages, similar to that of the overall participants. Conclusions: Our findings highlight the potential hazards of high protein intake on linear growth in children and adolescents. Caution should be exercised when promoting increased protein consumption in children and adolescents who already have a high intake of protein.

## 1. Introduction

Childhood and adolescence are critical periods for linear growth, which refers to the increase in body height. According to the Child Growth Standards established by the World Health Organization (WHO), individuals whose height falls below two standard deviations of the expected height for their gender and age are considered to have stunting [1]. This condition affects approximately one-quarter of children worldwide [2]. Stunted children and adolescents face a higher risk of mortality, morbidity, and suboptimal cognitive and motor development [3]. Consequently, stunting is recognized as one of the six global nutrition targets endorsed by the WHO to improve child nutrition by 2025 [4]. Therefore, it is crucial to maximize the potential for linear growth and reduce the occurrence of stunting in children and adolescents.

It is widely recognized that nutritional status directly impacts the growth and development of children and adolescents. Protein, which is an essential macronutrient, plays a crucial role in linear growth by meeting the metabolic demand for amino acids needed for tissue growth. Additionally, it elevates hormone levels such as insulin and insulin-like growth factor 1 (IGF-1), which promotes linear growth by stimulating endochondral ossification [5]. Protein deficiency has been proven to lead to significant decreases in IGF-1, both in animal [6] and human [7] studies, and this growth-limiting effect can be neutralized by increasing dietary protein intake [6]. Therefore, there is clear evidence that protein deficiency hampers linear growth. Interestingly, current evidence suggests that the protein intake levels in most developed countries exceed their dietary protein recommendations [8,9]. This trend is even more pronounced among children and adolescents, with their protein intakes being 2- to 3-fold higher than the recommended levels in many developed countries [10]. In China, for instance, 52.4% of children and adolescents have daily protein intakes that meet or exceed the dietary protein recommendations. Moreover, the ratios are significantly higher in urban areas compared to rural areas [11]. All of the evidence indicate that a considerable number of children and adolescents consume protein beyond the recommended levels worldwide. However, there is limited research focusing on the association between high protein intake and linear growth within this population, particularly considering the prevailing trend of high dietary protein consumption in many developed countries and many cities of China.

In this context, we aimed to explore the association between protein intake and linear growth in a population with relatively high protein intake in a modern metropolis on the southeast coast of China. Considering the critical impact of gender and puberty stage on the correlation between protein intake and linear growth, we also examined this association in different pubertal stages across genders within the same population.

## 2. Material and Methods

### 2.1. Study Participants

The study participants were from the Evaluation and Monitoring on School-based Nutrition and Growth in Shenzhen (EMSNGS). The EMSNGS study is an ongoing cohort study conducted in Shenzhen, a modern metropolis on the southeast coast of China. Its objective is to assess and monitor the nutritional status, growth and development, and other health outcomes in children and adolescents. The baseline survey for this study was completed in December 2021.

To select the participants, a multistage stratified cluster random sampling method was employed. Primary and secondary schools in ten administrative districts of the city were sampled. The sampling process consisted of two stages. In the first stage, one primary school, one junior high school, and one senior high school were chosen from each district, resulting in a total of 27 schools (including three nine-year schools). In the second stage, one class from each grade (excluding classes with fewer than 40 students) was randomly selected from each school. Overall, 112 classes were included, with a final participant count of 5153 school students.

All study participants and their guardians were informed about the study’s protocol and provided informed consent before enrollment. The study received approval from the ethics review committee of the Shenzhen Center for Chronic Disease Control (SZCCC-2021-037-01-PJ) and was registered at the China Clinical Trials Registry (ChiCTR2100051722; www.chictr.org.cn, accessed on 1 January 2022).

For the analysis, certain participants were excluded based on specific criteria (see Supplementary Figure S1). Initially, 35 participants without basic information, 129 participants with diseases that could affect growth, 44 participants without available disease data, and 15 participants taking medications that might impact growth were excluded. Additionally, we excluded 1361 individuals without dietary data (food frequency questionnaires were not conducted for students in grades 1 through 3, totaling 1359) and those with implausible total energy intake (total daily energy intake less than 0.5 times or greater than 2 times the estimated energy requirements of Chinese residents of different ages, totaling 173), non-fasting participants (totaling 84), participants without height data (totaling 2), and participants aged below 6 or above 18 years old (totaling 13). Ultimately, the analysis included 3299 students.

### 2.2. Dietary Assessment

Dietary intake was collected using a validated Food Frequency Questionnaire (FFQ) consisting of 61 food items, carefully considering the eating habits and food composition specific to Chinese individuals [12]. Experienced nutritionists provided guidance to participants, who estimated their food intake using food models representing standard portion sizes and food pictures depicting different portion sizes for all food items. Participants were asked to report their usual frequency of consuming each food item and the average serving size over the past month. The calculation of total energy intake and nutrient values relied primarily on the China Food Composition Database (6th edition), which served as the foundation for the food composition database [13]. To account for individual variations in energy intake, protein intake and other dietary nutrients were adjusted using the nutrient residual method [14]. Adjusting for total energy intake has been demonstrated to mitigate the impact of measurement errors inherent in FFQs when assessing protein intake. Furthermore, in this study, dietary protein intake was calculated per kilogram of body weight by incorporating energy-adjusted protein intake and body weights. This methodology has been previously validated in other studies [15]. As a result, protein intake was expressed as grams per kilogram of body weight per day (g·kg^−1^·d^−1^), as well as a percentage of total energy intake (%E).

### 2.3. Outcome Assessment

The primary outcomes included body height, height-for-age Z score (HAZ), and the prevalence of stunting. Qualified doctors and nurses who underwent rigorous training conducted the measurements of each participant’s height and weight. Participants were required to wear loose clothing, be barefoot, feel comfortable, and extend both legs during the measurements. Height was recorded to the nearest 0.1 cm, and weight to the nearest 0.1 kg. HAZ was calculated using the gender- and age-specific growth standards provided by WHO in 2006 [16]. According to the WHO Child Growth Standards, stunting is defined as a HAZ value below −2 [1].

### 2.4. Covariates

Information regarding age, gender, medical history, medication usage, lifestyle, family income, parental height, and education level was collected through structured questionnaires. Central obesity was defined as a waist circumference equal to or greater than the 90th percentile specific to age and gender [17]. A pediatrician from the endocrinology department assessed pubertal development stages based on Tanner stages (Tanner stage I–V), categorizing them as pre-puberty (Tanner stage I), mid-puberty (Tanner stage II–III), and post-puberty (Tanner stage IV or higher) [18,19,20]. Parents’ educational levels were classified as ≤9, 10–12, 13–15, and ≥16 years of completed schooling. Household income levels were categorized as <120,000, 120,000–249,999, and ≥250,000 Chinese yuan (CNY) (1 CNY ≈ 0.13 EUR; 1 CNY ≈ $ 0.14 USD), based on the average yearly household income. Nighttime sleep duration was determined by calculating the time spent sleeping at night until waking up in the morning. Moderate to vigorous physical activity (MVPA) was classified as <1.0, 1.0–2.9, and ≥3 h per day. Smoking status was categorized as never or yes, including <1 cigarette per month, ≥1 cigarette per month, and e-cigarette use. Drinking status was categorized as never, <1 standard drink per month, and ≥1 standard drink per month.

### 2.5. Statistical Analysis

All covariates were assessed for normality and analyzed using nonparametric methods if necessary. Descriptive statistics at baseline included means ± standard deviations (SDs) for continuous variables and percentages for categorical variables. Associations between participant characteristics, protein intake, and outcome variables were examined using the χ^2^ test for categorical variables and analysis of variance, and the Kruskal–Wallis rank test for continuous variables. Spline smoothing [21] was used to visually depict the dose–response relationship between protein intake and linear growth, as well as the rate of stunting.

Generalized linear models were employed to investigate the association between protein intake and height or height-for-age Z score (HAZ). Results were presented as coefficients (β) with 95% confidence intervals (CIs). Logistic regression analyses were conducted to calculate odds ratios (ORs) and 95% CIs, assessing the relationship between the prevalence of stunting and protein intake. Protein intake was evaluated as either a continuous variable (per 1 SD increase) or a categorical variable (quartiles of protein intake). Quartile 1 (Q1) was chosen as the reference group in each model when examining protein intake by quartiles. The linear trend across quartiles of protein intake was tested by assigning the median value for each quartile and analyzing it as a continuous variable in multivariate models. Protein intake was expressed in grams per kilogram weight per day (g·kg^−1^·d^−1^), as well as a percentage of total energy intake (%E).

Covariates associated with protein intake and linear growth, as reported in previous studies, were included as potential confounders in the analyses. In the association of characteristics of study participants with protein intake and outcomes, confounders such as serum vitamin D_3_, serum Zn, serum vitamin A, dietary fat intake, dietary carbohydrate intake, and mothers’ age were grouped according to tertiles. The following regression models were used: crude model (unadjusted); Model I, which was adjusted for gender, ethnicity, age (as a continuous variable), pubertal stages, premature, birth length, maternal age (as a continuous variable), parental education levels (as a continuous variable), parental height (as a continuous variable), household income levels, smoking status, drinking status, moderate to vigorous physical activity (MVPA), central obesity, length of nighttime sleep (as a continuous variable), dietary fat intake (as a continuous variable), and dietary carbohydrate intake (as a continuous variable); and Model II with additional adjustment for serum calcium, zinc, vitamin D_3_, and vitamin A levels, all as continuous variables. The premature status was missing for some of the participants (2/3299). We included the two participants with missing information on premature status in a separate missing data group in our main analysis. Similar procedures were applied to other missing variables, including household income, MVPA, length of nighttime sleep, fathers’ height, fathers’ education, and mothers’ education.

To assess potential modifiers of linear growth, such as gender (male or female) and pubertal stages (pre-puberty, mid-puberty, post-puberty), stratified analyses were conducted, and *p* values for interaction were estimated using the log-likelihood ratio test. Analyses were also performed separately for animal and plant protein intake in relation to body height and HAZ.

All statistical analyses were conducted using Empower (R) software based on R (version 3.4.3) from X&Y Solutions, Inc., Boston, MA, USA. Significance was determined at two-tailed *p* values of < 0.05 for all analyses.

## 3. Results

### 3.1. Characteristics of the Participants

A total of 3299 participants were included in the study, and Table 1 presents their characteristics. The mean protein intake was 1.91 ± 0.64 g·kg^−1^·d^−1^ (17% ± 6%). The prevalence of stunting was 1.58% (52/3299). Protein intake, assessed in grams per kilogram weight per day or as a percentage of total energy intake, was significantly associated with gender, age, drinking status, MVPA, central obesity, length of nighttime sleep, serum zinc, vitamin D_3_, and vitamin A levels, as well as fat and carbohydrate intake (all *p* < 0.05). When assessing protein intake in grams per kilogram weight per day, additional associations were observed with pubertal stage, serum calcium level, parental height, and parental educational levels (all *p* < 0.05). Boys had higher height (*p* < 0.001) and HAZ (*p* < 0.001) than girls, resulting in a higher prevalence of stunting among females. Taller participants were older and had lower protein intake, more time spent on MVPA, greater incidence of central obesity, longer length of nighttime sleep, higher serum calcium, zinc, vitamin D_3_, and vitamin A levels, taller parents, and higher parental educational levels. HAZ was associated with protein intake, age, household income levels, MVPA time, central obesity, length of nighttime sleep, vitamin D_3_ level, serum calcium level, parental height, and parental educational levels. The prevalence of stunting was associated with gender, age, ethnicity, paternal height, MVPA time, central obesity, protein intake, and parental educational levels. More details are provided in Table 1.

### 3.2. Association of Protein Intake with Linear Growth in Children and Adolescents

**Figure 1:**
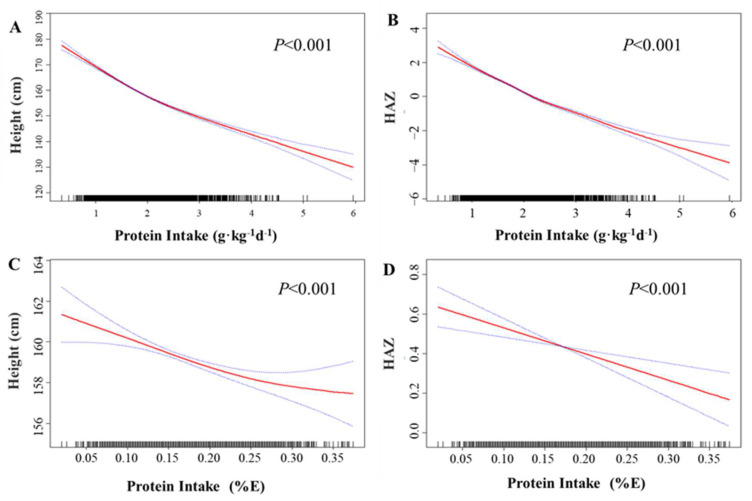
The association between protein intake and linear growth. Spline smoothing, based on a generalized additive model, was adjusted for gender ethnicity, age (as a continuous variable), pubertal stages, premature, birth length (as a continuous variable), maternal age (as a continuous variable), parental education levels (as a continuous variable), parental height (as a continuous variable), household income levels, smoking status, drinking status, moderate to vigorous physical activity, central obesity, length of nighttime sleep (as a continuous variable), dietary fat intake (as a continuous variable), dietary carbohydrate intake (as a continuous variable), and serum calcium, zinc, vitamin D_3_, and vitamin A levels (as continuous variables). (**A**) The association between protein intake (g·kg^−1^·d^−1^) with body height; (**B**) The association between protein intake (g·kg^−1^·d^−1^) with HAZ; (**C**) The association between protein intake (E%) with body height; (**D**) The association between protein intake (E%) with body height.

Figure 1 illustrates the dose–response relationship between protein intake and linear growth of the participants. In the fully adjusted Model II, a significantly negative association was observed between protein intake (g·kg^−1^·d^−1^) and body height (Figure 1A) (*p* < 0.001), as well as HAZ (Figure 1B) (*p* < 0.001). A similar relationship was found between protein intake (%E) and body height (Figure 1C) (*p* < 0.001) and HAZ (Figure 1D) (*p* < 0.001). Results from the generalized linear model (Table 2) indicated that each unit increase in protein intake (g·kg^−1^·d^−1^) was associated with a change in body height by −9.45 cm (95% CI: −9.72, −9.18) and a change in HAZ by −0.21 (95% CI: −0.25, −0.18) in the crude model. After adjusting for participant demographics, lifestyle factors, and paternal factors in Model I, each unit increase in protein intake was found to be associated with a change in height by −5.79 cm (95% CI: −6.12, −5.45) and a change in HAZ by −0.78 (95% CI: −0.83, −0.73). Further adjustment for serum calcium, zinc, vitamin D_3_, and vitamin A levels in Model I yielded consistent results, with each unit increase in protein intake (g·kg^−1^·d^−1^) linked to a change in linear growth by −5.78 cm (95% CI: −6.12, −5.45) and a change in HAZ by −0.79 (95% CI: −0.84, −0.74). Similar results were obtained when assessing protein intake as a percentage of total energy intake (%E) (Table 2). We also separately examined animal and plant protein intake, both of which showed negative associations with linear growth, similar to the overall negative relationship with total protein intake (Supplementary Figure S2 and Supplementary Table S1).

Considering the possible impact of gender and puberty stage on the correlation between protein intake and linear growth, we conducted further analysis on the association between dietary protein intake and linear growth in different pubertal stages across different genders and found a similar significant negative correlation. More details are provided in Table 3.

In addition, to assess potential modifiers of linear growth, such as gender (boy or girl) and pubertal stages (pre-puberty, mid-puberty, post-puberty), stratified analyses were conducted. The relationship between protein intake, linear growth, and HAZ remained consistent in stratified analyses by gender and pubertal stages. However, we observed that the association between dietary protein intake and height was more pronounced in males (*p*_for interaction_ < 0.001) and those in the post-puberty stage (*p*_for interaction_ < 0.001) (Supplementary Figures S3 and S4 and Supplementary Table S2).

## 4. Discussion

In a large cross-sectional study using multistage stratified cluster random sampling, we observed for the first time a consistent negative association between protein intake and linear growth in children and adolescents after adjusting for potential confounders. Moreover, we found that higher protein intake was associated with a higher prevalence of stunting.

While previous studies have primarily focused on body weight, body composition, and related outcomes such as body mass index or fat mass index [10], our study specifically examined linear growth and its association with protein intake. Our findings contradict two earlier cross-sectional studies that reported a positive association between dietary protein intake and linear growth [21,22]. Some prospective studies also reported a positive association between protein intake with height [23,24,25,26]. For instance, Catherine S Berkey et al. contributed to research on the impact of milk on growth in a cohort of 5101 premenarchal girls from throughout the United States completed annual surveys (1996–2001, 2003). They found, that of the foods/nutrients studied, dairy protein had the strongest association with height growth. But the study finally suggested that the factor in the non-lipid phase of milk, but not the protein itself, has growth-promoting action in girls [23]. Aliya Alimujiang et al. designed their study for a different purpose, but it also reported a positive association between animal protein intake and height at age 13, as well as adult height [24]. It is noteworthy that the 229 participants were Caucasians of predominantly northern European descent from low- to middle-class families in Boston in the 1930s. The protein intake levels were not high. This was fundamentally different from the protein intake levels in our study. In contrast to our findings, Yifan Hua et al. also observed a direct relationship between protein intake and linear length in 189 girls [25]. However, their average protein intake, assessed as a percentage of total energy intake (%E), was between 12.5% and 13.3%. In our study, the participants were from Shenzhen, a modern metropolis on the southeast coast of China, where the average protein intake was as high as 17.0%, 1.81 g·kg^−1^·d^−1^, significantly higher than the aforementioned study. According to the Recommended Dietary Allowance in the US, the recommended protein intake for age groups of 4–13 years and 14–17 years is 0.95 g·kg^−1^·d^−1^ and 0.85 g·kg^−1^·d^−1^, respectively. Hence, the protein intake in our study significantly exceeded the recommended dietary protein intake in the US and closely resembled the current protein intake levels in children and adolescents in developed countries in Europe and the US [8]. Therefore, we speculate that the previously observed discrepancies in associations between protein intake and linear growth might be attributed to different average protein intake levels. However, Kim Ve Braun et al. conducted a study where protein intake, comparable to our study at 1.9 g/kg BW per day, still showed a significant association. They found that a 10-g higher total protein intake per day at 1 year was significantly associated with a greater height of 0.03 SD [26]. However, it was an early programming longitudinal study. As we know, individuals at a younger age have a greater demand for protein; therefore, a higher protein intake may not necessarily exert negative effects on early linear growth. However, two other studies did not find any significant association between protein intake and linear growth [23,27].

Therefore, the discrepancies may be attributed to variations in protein intake assessment methods (grams per day, grams per kilogram of body weight, or as a percentage of total energy intake), types of proteins studied (total proteins, animal proteins, plant proteins, etc.), and average protein intake levels. In this study, we primarily calculated protein intake in grams per kilogram of body weight to minimize any confounding effects of body weight status. Additionally, we assessed protein intake as a percentage of total energy intake (%E) to investigate whether different ways of measuring protein intake could explain previously observed discrepancies in associations with linear growth. We found a similar but weaker association between protein intake and linear growth when assessing protein intake as a percentage of total energy intake instead of grams per kilogram of body weight. Furthermore, we examined protein intake from both animal and plant sources separately, and both showed negative associations with body height and HAZ, similar to the associations observed with total protein intake. Therefore, neither the different methods of assessing protein intake nor the specific types of proteins accounted for the discrepancies between our study and previous studies.

In addition, a large population-based study in the US, which had relatively high protein intake, found that elevated protein levels during pregnancy were associated with shorter offspring birth length and slower linear growth into mid-childhood [15], and these findings align with our study results.

Although the precise molecular mechanisms underlying the relationship are unclear, our results are biologically plausible. On the one hand, a recent study demonstrated that a high maternal protein diet can impair offspring’s bone mass through miR-24-1-5p-mediated targeting of recombinant SMAD family member 5 (SMAD5) [28]. SMAD5 is a well-characterized downstream component of the bone morphogenetic protein signaling pathway in osteoblasts, and its impairment leads to reduced osteoblast maturation and diminished bone mass [29]. This finding suggests that high protein intake may negatively affect osteoblast maturation and bone mass. Therefore, this study provides a potential explanation for our results, pending confirmation in children and adolescents in future research. On the other hand, excessive protein intake can lead to an abundance of amino acids, which may accelerate amino acid oxidation and result in toxic levels of ammonia in the plasma. Ultimately, this could potentially hinder linear growth [30].

The strength of our study lies in being the first to observe a negative association between high protein intake and linear growth in children and adolescents, drawing more attention to this issue. Moreover, we assessed protein intake both as grams per kilogram of body weight and as a percentage of total energy intake (%E), which helps eliminate the influence of different methods of protein intake evaluation on the results. Furthermore, we adjusted for numerous confounders such as pubertal development stages, serum vitamin D_3_ levels, serum vitamin A levels, serum calcium and zinc levels, physical exercise intensity, birth outcomes, parental height, etc., thus minimizing the impact of confounding factors on our results. Lastly, the large sample size of our study allowed us to conduct stratified analyses.

However, there are several limitations in our study. It is a cross-sectional study, making it difficult to establish causal relationships. Additionally, dietary assessments were performed using the FFQ, and although extensively validated in various populations [12], there may be some measurement error in estimating absolute nutrient intake. Nevertheless, we employed multiple approaches to address this error. Firstly, consistent results were obtained when ranking participants into quartiles of protein intake and when analyzing protein intake as a continuous variable. This method is considered valid for analyzing data obtained from FFQs [14]. Furthermore, adjusting for total energy intake is a common technique for improving the validity of FFQs [14], and our nutrient intake estimates were all energy-adjusted using the nutrient residual method.

## 5. Conclusions

In conclusion, this large cross-sectional study in children and adolescents revealed that higher protein intake was associated with shorter linear growth and a higher prevalence of stunting. These findings suggest that increased dietary protein intake among well-nourished children and adolescents may not only fail to promote linear growth, but also impair it. Therefore, considering the potential risks of excessive protein intake is important. More studies are needed to confirm the causal relationship between them in the future.

## Figures and Tables

**Table 1:** Association of characteristics of study participants with protein intake and outcomes (N = 3299).

Variables	n	Protein Intake, g·kg^−1^·d^−1^		Protein Intake, %E		Height (cm)		HAZ		Stunning	
		Mean ± SD	*p*	Mean ± SD	*p*	Mean ± SD	*p*	Mean ± SD	*p*	N (%)	*p*
Overall	3299										
Basic characteristics											
Gender			<0.001		<0.001		<0.001		<0.001		0.025
Male	1837	1.82 ± 0.66		15% ± 5%		161.76 ± 13.63		0.55 ± 1.00		21 (1.14)	
Female	1462	2.03 ± 0.60		19% ± 6%		155.86 ± 9.65		0.31 ±0.92		31 (2.12)	
Age, years			<0.001		<0.001		<0.001		<0.001		0.052
9.0–12.0	1074	2.44 ± 0.67		18% ± 6%		146.44 ± 8.48		0.74 ± 1.06		9 (0.84)	
12.0–14.0	849	1.85 ± 0.48		17% ± 5%		160.44 ± 7.85		0.61 ± 1.04		18 (2.12)	
>14.0	1376	1.54 ± 0.37		17% ± 5%		168.16 ± 8.04		0.11 ± 0.87		25 (1.82)	
Ethnicity			0.976		0.428		0.373		0.303		<0.001
Han Chinese	3177	1.91 ± 0.64		17% ± 5%		159.18 ± 12.41		0.44 ± 0.97		45 (1.42)	
Others	122	1.91 ± 0.62		17% ± 6%		158.16 ± 11.68		0.35 ± 1.07		7 (5.74)	
Premature			0.279		0.558		0.115		0.996		0.543
Yes	3123	1.91 ± 0.64		17% ± 5%		159.24 ± 12.41		0.44 ± 1.02		51 (1.63)	
No	174	1.97 ± 0.62		17% ± 6%		156.53 ± 11.58		0.44 ± 0.98		1 (0.57)	
Missing	2	1.40 ± 0.30		14% ± 8%		165.00 ± 2.69		0.50 ± 0.71		0 (0.00)	
Birth length, cm			<0.001		0.330		<0.001		<0.001		0.336
Low	651	2.02 ± 0.64		17% ± 5%		156.78 ± 11.96		0.28 ± 1.01		13 (2.00)	
High	2648	1.89 ± 0.64		17% ± 6%		159.67 ± 12.41		0.48 ± 1.02		39 (1.47)	
Pubertal stage					0.667		<0.001		<0.001		0.815
Pre-puberty	343	2.68 ± 0.71	<0.001	17% ± 5%		141.37 ± 6.61		0.42 ± 0.91		5 (1.46)	
Mid-puberty	887	2.26 ± 0.65		17% ± 5%		151.04 ± 9.72		0.64 ± 1.06		16 (1.80)	
Post-puberty	2069	1.64 ± 0.41		17% ± 6%		165.57 ± 8.66		0.36 ± 0.93		31 (1.50)	
Household income, CNY/years			0.569		0.929		0.720		<0.001		0.372
<120,000	935	1.90 ± 0.62		17% ± 6%		158.84 ± 11.95		0.30 ± 0.97		19 (2.03)	
120,000–250,000	1126	1.90 ± 0.66		17% ± 6%		159.38 ± 12.18		0.43 ± 0.98		19 (1.69)	
≥250,000	1214	1.93 ± 0.64		17% ± 5%		159.13 ± 12.87		0.56 ± 0.95		14 (1.15)	
Missing	24	1.87 ± 0.77		17% ± 5%		160.67 ± 13.78		0.18 ± 0.86		0 (0.00)	
Smoking status			0.195		0.797		<0.001		0.061		0.344
no	3244	1.91 ± 0.64		17% ± 6%		158.99 ± 12.31		0.44 ± 1.02		52 (1.60)	
yes	55	1.80 ± 0.75		17% ± 6%		165.88 ± 14.14		0.22 ± 1.03		0 (0.00)	
Drinking status, standard drink/month			<0.001		<0.001		<0.001		0.109		0.166
Never	2819	1.96 ± 0.65		17% ± 5%		158.16 ± 12.35		0.46 ± 0.98		43 (1.53)	
<1	369	1.67 ± 0.53		16% ± 6%		163.99 ± 11.11		0.34 ± 0.90		9 (2.44)	
≥1	111	1.54 ± 0.39		16% ± 6%		168.19 ± 9.67		0.33 ± 0.93		0 (0.00)	
MVPA, h/day			<0.001		<0.001		<0.001		<0.001		0.035
<1.0	770	1.79 ± 0.56		18% ± 6%		161.16 ± 11.00		0.21 ± 1.00		19 (2.47)	
1–3	1505	1.88 ± 0.61		17% ± 5%		159.91 ± 12.05		0.46 ± 1.01		21 (1.40)	
>3	1010	2.04 ± 0.72		16% ± 5%		156.42 ± 13.29		0.60 ± 1.02		11 (1.09)	
Missing	14	2.26 ± 0.96		18% ± 6%		151.99 ± 16.03		0.07 ± 1.27		1 (7.14)	
Central obesity			<0.001		0.028		<0.001		<0.001		0.018
No	2784	2.01 ± 0.63		17% ± 5%		158.47 ± 12.41		0.32 ± 0.96		50 (1.80)	
Yes	515	1.40 ± 0.41		17% ± 6%		162.50 ± 11.62		1.09 ± 1.09		2 (0.39)	
Length of nighttime sleep, h/day			<0.001		<0.001		<0.001		<0.001		0.824
<8	635	1.52 ± 0.40		17% ± 6%		167.93 ± 8.69		0.11 ± 0.88		11 (1.73)	
8–9	1205	1.72 ± 0.49		17% ± 5%		163.91 ± 9.67		0.39 ± 1.00		21 (1.74)	
≥9	1442	2.25 ± 0.68		18% ± 6%		151.19 ± 10.97		0.64 ± 1.05		20 (1.39)	
Missing	16	1.88 ± 0.72		16% ± 4%		159.47 ± 15.31		0.00 ± 0.89		0 (0.00)	
Protein intake, g·kg^−1^·d^−1^			<0.001		<0.001		<0.001		<0.001		<0.001
Q1	825	1.22 ± 0.19		14% ± 5%		170.23 ± 8.24		0.66 ± 1.00		3 (0.36)	
Q2	824	1.64 ± 0.09		17% ± 5%		163.60 ± 8.18		0.50 ± 0.94		5 (0.61)	
Q3	825	2.00 ± 0.12		18% ± 6%		157.09 ± 8.44		0.43 ± 0.99		18 (2.18)	
Q4	825	2.80 ± 0.49		18% ± 5%		145.66 ± 8.86		0.17 ± 0.88		26 (3.15)	
Biochemical indexes											
Serum vitamin D_3_, ng/mL			<0.001		0.002		<0.001		<0.001		0.383
T1	1093	1.76 ± 0.54		18% ± 6%		162.38 ± 10.49		0.29 ± 0.95		15 (1.37)	
T2	1093	1.89 ± 0.61		17% ± 6%		159.54 ± 11.94		0.50 ± 1.04		22 (2.01)	
T3	1094	2.08 ± 0.72		17% ± 5%		155.54 ± 13.49		0.53 ± 1.05		15 (1.37)	
Serum Ca, mmol/L			<0.001		0.594		<0.001		<0.001		0.745
Low	697	1.79 ± 0.55		17% ± 5%		162.11 ± 10.99		0.33 ± 0.92		12 (1.72)	
High	2584	1.94 ± 0.66		17% ± 6%		158.40 ± 12.60		0.47 ± 0.98		40 (1.55)	
Serum Zn, mg/L			0.048		0.015		0.003		0.919		0.582
T1	1092	1.95 ± 0.65		17% ± 6%		158.21 ± 12.25		0.45 ± 0.97		14 (1.28)	
T2	1094	1.89 ± 0.64		17% ± 5%		159.41 ± 12.37		0.43 ± 0.98		20 (1.83)	
T3	1093	1.89 ± 0.64		17% ± 5%		159.97 ± 12.42		0.44 ± 0.97		18 (1.65)	
Serum vitamin A, μg/mL			<0.001		<0.001		<0.001		0.181		0.666
T1	1087	2.10 ± 0.68		18% ± 6%		155.48 ± 12.27		0.40 ± 0.93		20 (1.84)	
T2	1091	1.95 ± 0.61		17% ± 6%		158.45 ± 11.76		0.44 ± 0.99		17 (1.56)	
T3	1102	1.69 ± 0.56		16% ± 5%		163.59 ± 11.68		0.48 ± 0.99		15 (1.36)	
Dietary fat intake (g/d)			<0.001		<0.001		<0.001		0.137		0.241
T1	1100	1.69 ± 0.56		15% ± 5%		160.16 ± 13.03		0.48 ± 0.98		14 (1.27)	
T2	1099	1.97 ± 0.61		19% ± 5%		157.88 ± 12.02		0.40 ± 0.97		15 (1.36)	
T3	1100	2.08 ± 0.69		18% ± 5%		159.40 ± 11.97		0.44 ± 0.97		23 (2.09)	
Dietary carbohydrate intake (g/d)			<0.001		<0.001		<0.001		0.040		0.431
T1	1100	2.11 ± 0.69		18% ± 5%		159.36 ± 12.07		0.46 ± 0.98		19 (1.73)	
T2	1099	1.95 ± 0.60		19% ± 5%		158.05 ± 11.95		0.38 ± 0.97		20 (1.82)	
T3	1100	1.67 ± 0.55		14% ± 5%		160.02 ± 13.03		0.48 ± 0.96		13 (1.18)	
Parental characteristics											
Fathers’ height, cm			0.047		0.283		<0.001		<0.001		<0.001
Q1	680	1.96 ± 0.67		17% ± 6%		157.01 ± 12.48		−0.00 ± 0.92		31 (4.56)	
Q2	1031	1.93 ± 0.64		17% ± 6%		158.44 ± 12.01		0.27 ± 0.90		14 (1.36)	
Q3	721	1.89 ± 0.60		17% ± 5%		159.79 ± 11.73		0.61 ± 0.90		2 (0.28)	
Q4	824	1.88 ± 0.65		17% ± 5%		161.14 ± 13.00		0.87 ± 0.94		4 (0.49)	
Missing	43	1.78 ± 0.68		17% ± 6%		160.93 ± 11.78		0.47 ± 0.90		1 (2.33)	
Mothers’ height, cm			<0.001		0.158		<0.001		<0.001		<0.001
Q1	834	1.99 ± 0.66		17% ± 6%		156.44 ± 11.97		0.05 ± 0.92		29 (3.48)	
Q2	674	1.95 ± 0.63		17% ± 5%		157.51 ± 12.09		0.29 ± 0.91		12 (1.78)	
Q3	939	1.87 ± 0.63		17% ± 6%		160.56 ± 12.03		0.54 ± 0.93		8 (0.85)	
Q4	827	1.85 ± 0.63		17% ± 5%		161.57 ± 12.71		0.85 ± 0.94		3 (0.36)	
Missing	25	2.12 ± 0.84		18% ± 6%		160.28 ± 13.37		0.28 ± 0.86		0 (0.00)	
Fathers’ education, years			<0.001		0.557		0.021		<0.001		0.006
≤9 years	605	1.84 ± 0.57		17% ± 5%		160.19 ± 11.10		0.18 ± 0.97		17 (2.81)	
10~12 years	764	1.88 ± 0.64		17% ± 6%		159.61 ± 12.48		0.32 ± 0.97		16 (2.09)	
13~15 years	941	1.91 ± 0.62		17% ± 6%		158.82 ± 12.25		0.49 ± 0.97		13 (1.38)	
≥16 years	948	2.00 ± 0.70		17% ± 5%		158.33 ± 13.16		0.66 ± 0.93		5 (0.53)	
Missing	41	1.75 ± 0.67		17% ± 6%		161.44 ± 11.77		0.45 ± 0.89		1 (2.44)	
Mothers’ education, years			<0.001		0.182		<0.001		<0.001		<0.001
≤9 years	721	1.81 ± 0.56		17% ± 6%		160.19 ± 11.40		0.17 ± 0.98		23 (3.19)	
10~12 years	921	1.87 ± 0.62		17% ± 6%		159.88 ± 12.36		0.40 ± 0.97		16 (1.74)	
13~15 years	923	1.96 ± 0.66		17% ± 5%		158.81 ± 12.60		0.52 ± 0.97		12 (1.30)	
≥16 years	714	2.01 ± 0.70		17% ± 5%		157.44 ± 12.89		0.67 ± 0.90		1 (0.14)	
Missing	20	1.95 ± 0.78		18% ± 7%		163.55 ± 12.36		0.26 ± 0.80		0 (0.00)	
Mothers’ age, years			<0.001		0.818		<0.001		<0.001		0.350
T1	1044	2.12 ± 0.70		17% ± 5%		153.67 ± 12.05		0.55 ± 1.05		15 (1.44)	
T2	950	1.92 ± 0.60		17% ± 6%		159.64 ± 11.69		0.44 ± 1.01		12 (1.26)	
T3	1267	1.74 ± 0.56		17% ± 6%		163.11 ± 11.49		0.37 ± 0.99		25 (1.97)	

Abbreviations: HAZ, height-for-age z score; MVPA, moderate to vigorous physical activity; SD, standard deviation.

**Table 2:** Association of protein intake (per SD) with body height and HAZ.

Models	Protein Intake, g·kg^−1^·d^−1^				Protein Intake, %E			
	Body Height		HAZ		Body Height		HAZ	
	β (95%CI)	*p*	β (95%CI)	*p*	β (95%CI)	*p*	β (95%CI)	*p*
Crude model	−9.45 (−9.72, −9.18)	<0.001	−0.21 (−0.25, −0.18)	<0.001	−2.20 (−2.61, −1.78)	<0.001	−0.09 (−0.12, −0.05)	<0.001
Model I	−5.79 (−6.12, −5.45)	<0.001	−0.78 (−0.83, −0.73)	<0.001	−0.50 (−0.72, −0.28)	<0.001	−0.04 (−0.07, −0.01)	0.018
Model II	−5.78 (−6.12, −5.45)	<0.001	−0.79 (−0.84, −0.74)	<0.001	−0.48 (−0.70, −0.26)	<0.001	−0.04 (−0.07, −0.01)	0.014

Abbreviations: HAZ, height-for-age z score; SD, standard deviation. A generalized linear model was used. Protein intake was set to 1 SD increase of grams per kilogram weight per day (g·kg^−1^·d^−1^) or percentage of total energy intake (%E). Crude model, not adjusted for any variables; Model I, adjusted for gender, ethnicity, age (as a continuous variable), pubertal stages, premature, birth length (as a continuous variable), maternal (age as a continuous variable), parental education levels (age as a continuous variable), parental height (as a continuous variable), household income levels, smoking status, drinking status, moderate to vigorous physical activity (MVPA), central obesity, length of nighttime sleep (as a continuous variable), dietary fat intake (as a continuous variable), and dietary carbohydrate intake (as a continuous variable); Model II, additional adjustment for serum calcium, zinc, vitamin D_3_, and vitamin A levels as continuous variables.

**Table 3:** The association of protein intake (per SD) with body height and HAZ in different pubertal stages across different genders.

Pubertal Stage	Boys					Girls				
	n	Body Height		HAZ		n	Body Height		HAZ	
		β (95%CI)	*p*	β (95%CI)	*p*		β (95%CI)	*p*	β (95%CI)	*p*
Pre-puberty	266	−3.92 (−4.52, −3.32)	<0.001	−0.61 (−0.71, −0.50)	<0.001	77	−3.97 (−5.54, −2.40)	<0.001	−0.60 (−0.88, −0.31)	<0.001
Mid-puberty	580	−6.53 (−7.15, −5.91)	<0.001	−0.94 (−1.03, −0.84)	<0.001	307	−4.69 (−5.45, −3.93)	<0.001	−0.77 (−0.90, −0.64)	<0.001
Post-puberty	991	−5.82 (−6.55, −5.08)	<0.001	−0.73 (−0.83, −0.62)	<0.001	1078	−4.79 (−5.38, −4.20)	<0.001	−0.69 (−0.79, −0.60)	<0.001

Abbreviations: HAZ, height-for-age z score; SD, standard deviation. A generalized linear model was used. Protein intake was set to 1 SD increase of grams per kilogram weight per day (g·kg^−1^·d^−1^). Adjusted for ethnicity, age (as a continuous variable), pubertal stages, premature, birth length (as a continuous variable), maternal age (as a continuous variable), parental education levels (as a continuous variable), parental height (as a continuous variable), household income levels, smoking status, drinking status, moderate to vigorous physical activity (MVPA), central obesity, length of nighttime sleep (as a continuous variable), dietary fat intake (as a continuous variable), and dietary carbohydrate intake (as a continuous variable), and serum calcium, zinc, vitamin D_3_, and vitamin A levels as continuous variables.

## Data Availability

Data described in the manuscript, code book, and analytic code will be made available upon request pending reasonable application and approval.

## Supplementary Materials

The following supporting information can be downloaded at: https://www.mdpi.com/article/10.3390/nu15224821/s1, Figure S1: Flow of participants in current study.; Figure S2: The association of different sources of protein intake from with body height and HAZ.; Figure S3: The gender-specific association between dietary protein intake and linear growth.; Figure S4: The puberty stage-specific association between dietary protein intake and linear growth.; Table S1: Association of protein intake from different sources with body height and HAZ.; Table S2. Stratified analyses of potential modification effect for the association of dietary protein intake on linear growth.

## Abbreviations

BMI, body mass index; CIs, confidence intervals; CNY, China Yuan; EMSNGS, Evaluation and Monitoring on School-based Nutrition and Growth in Shenzhen; FFQ, food frequency questionnaire; HAZ, height-for-age Z score; IGF-1, insulin and insulin-like growth factor 1; OR, odds ratio; SD, standard deviation; WHO, World Health Organization.
