# Supplementary material for: Associations between High Protein Intake, Linear Growth, and Stunting in Children and Adolescents: A Cross-Sectional Study
Source: Nutrients. 2023 Nov 17;15(22):4821. doi: 10.3390/nu15224821 (PMC10675685; doi:10.3390/nu15224821)
Supplement: Supplementary file 1 [file nutrients-15-04821-s001.zip › nutrients-2685696-supplementary.pdf]

Associations between High Protein Intake, Linear Growth, and Stunting in Children and Adolescents: A Cross-Sectional Study

Supplementary online content

Supplemental Figure S1. Flow of participants in current study.

Supplemental Figure S2. The association of different sources of protein intake with body height and HAZ.

Supplemental Figure S3. The gender-specific association between dietary protein intake and linear growth.

Supplemental Figure S4. The puberty stage-specific association between dietary protein intake and linear growth.

Supplemental Table S1. Association of protein intake from different sources with body height and HAZ.

Supplemental Table S2. Stratified analyses of potential modification effect for the association of dietary protein intake with linear growth.

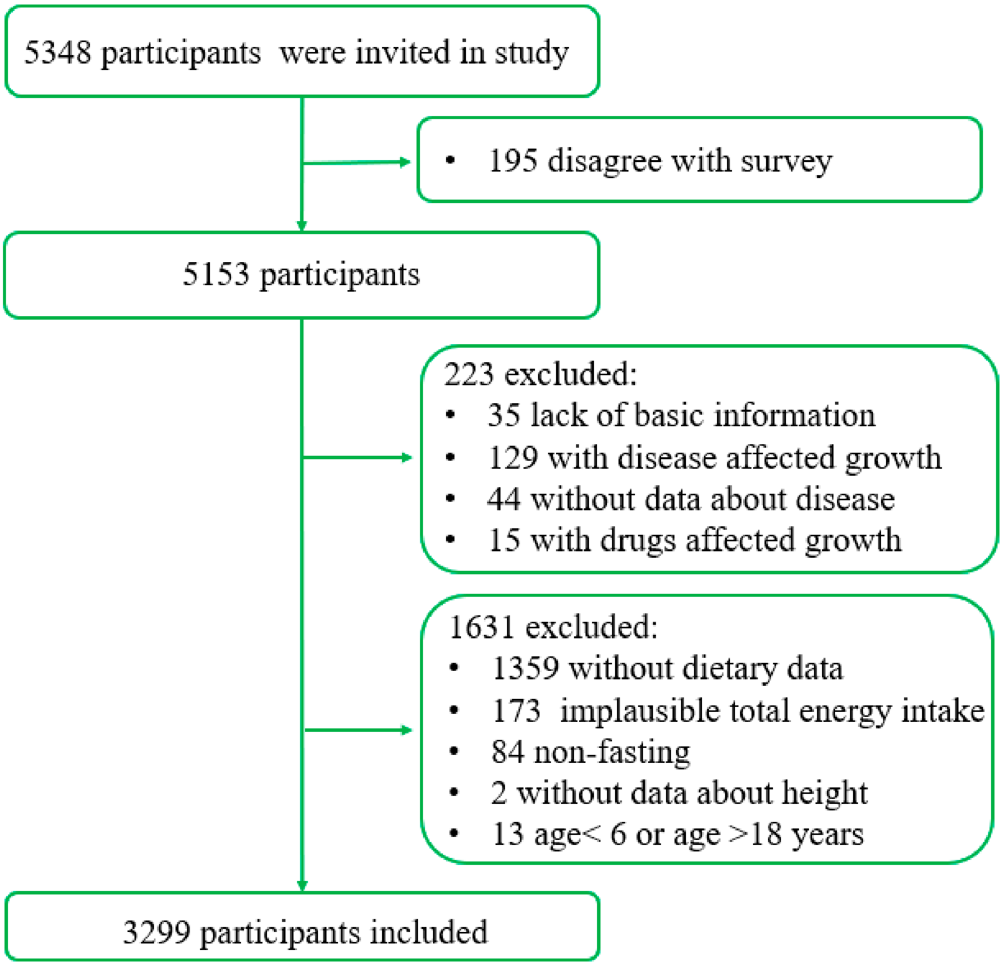

Supplemental Figure S1. Flow of participants in current study

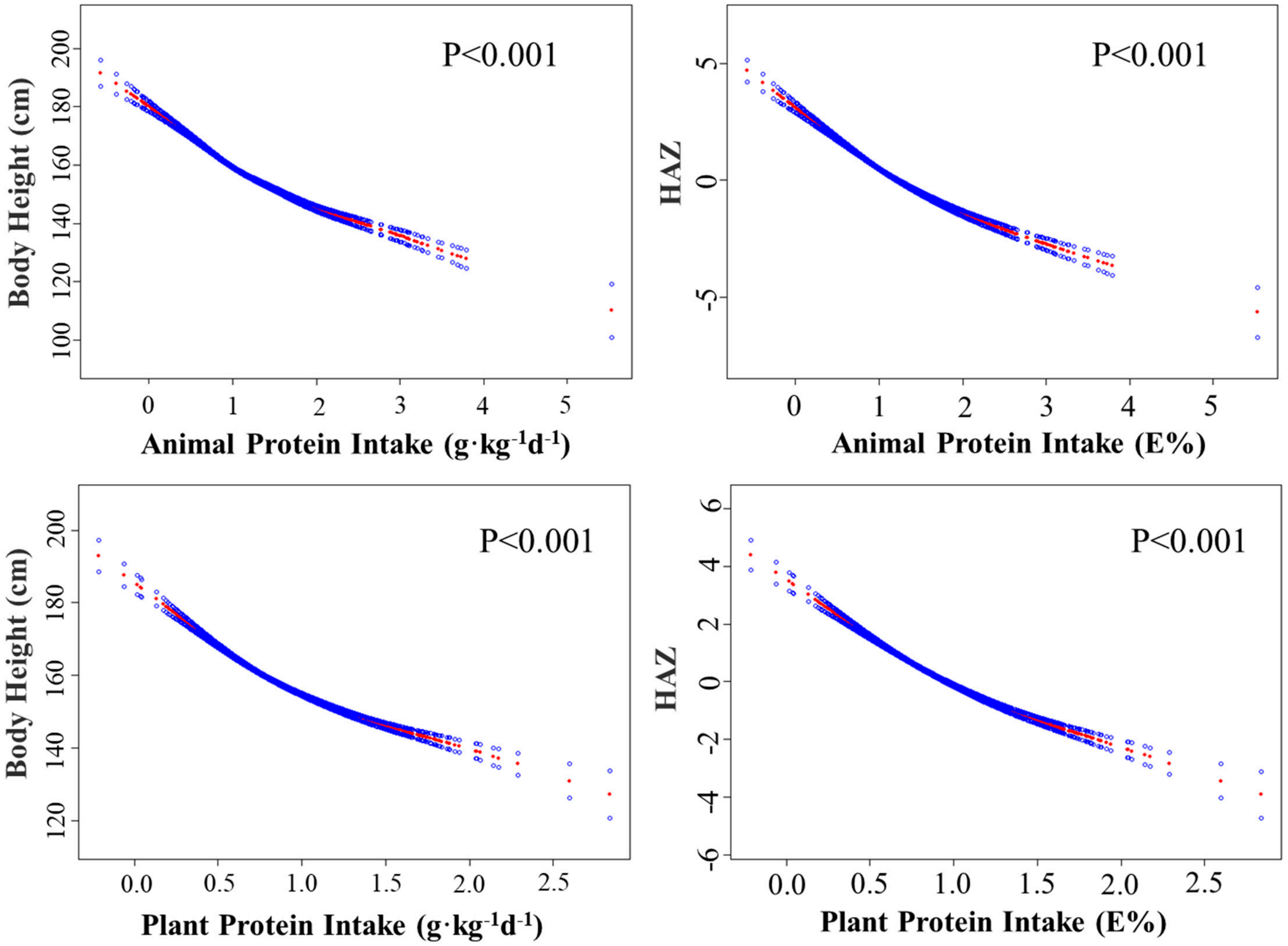

Supplemental Figure S2. The association of different sources of protein intake with body height and HAZ.

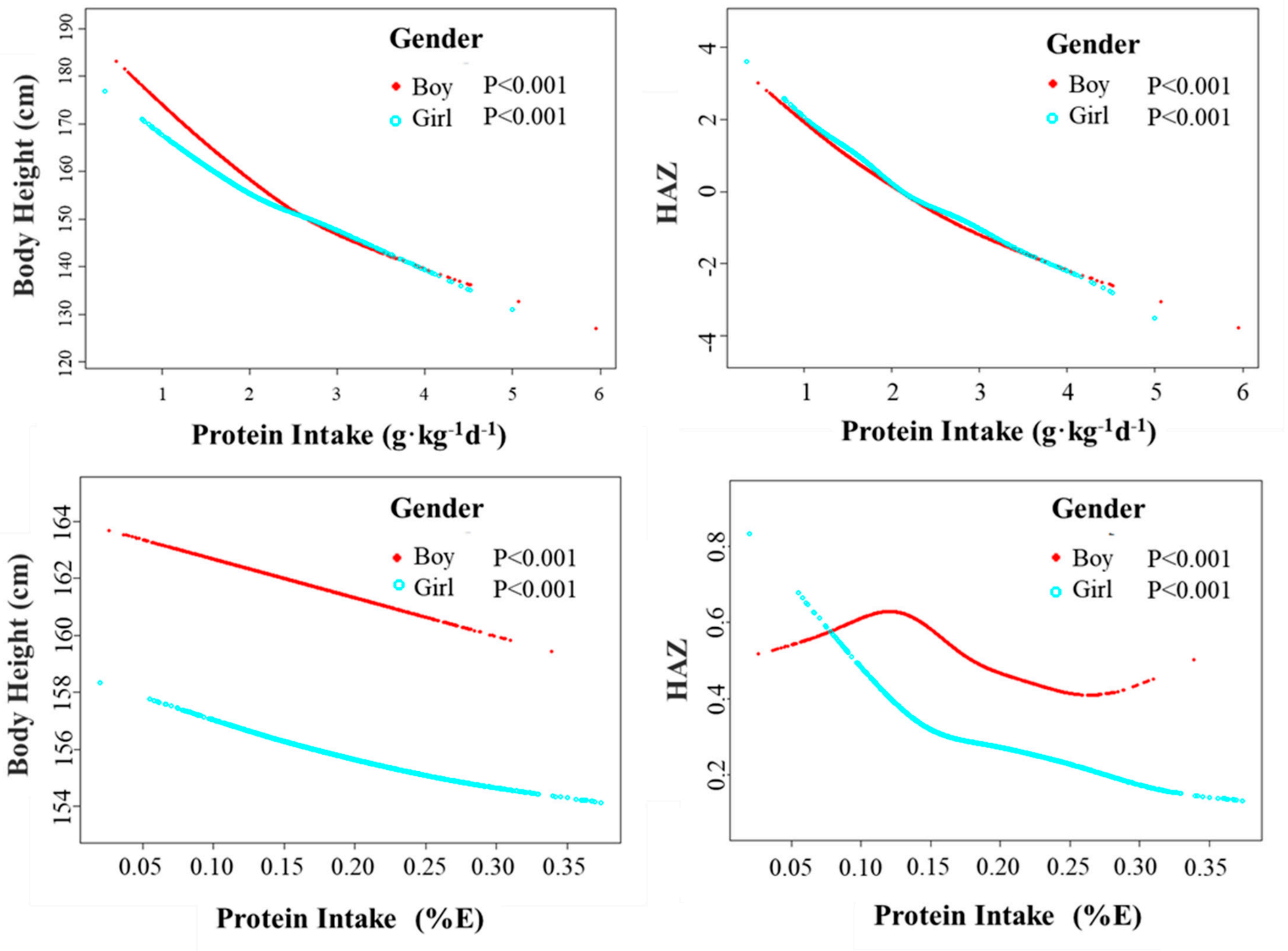

Supplemental Figure S3. The gender-specific association between dietary protein intake and linear growth.

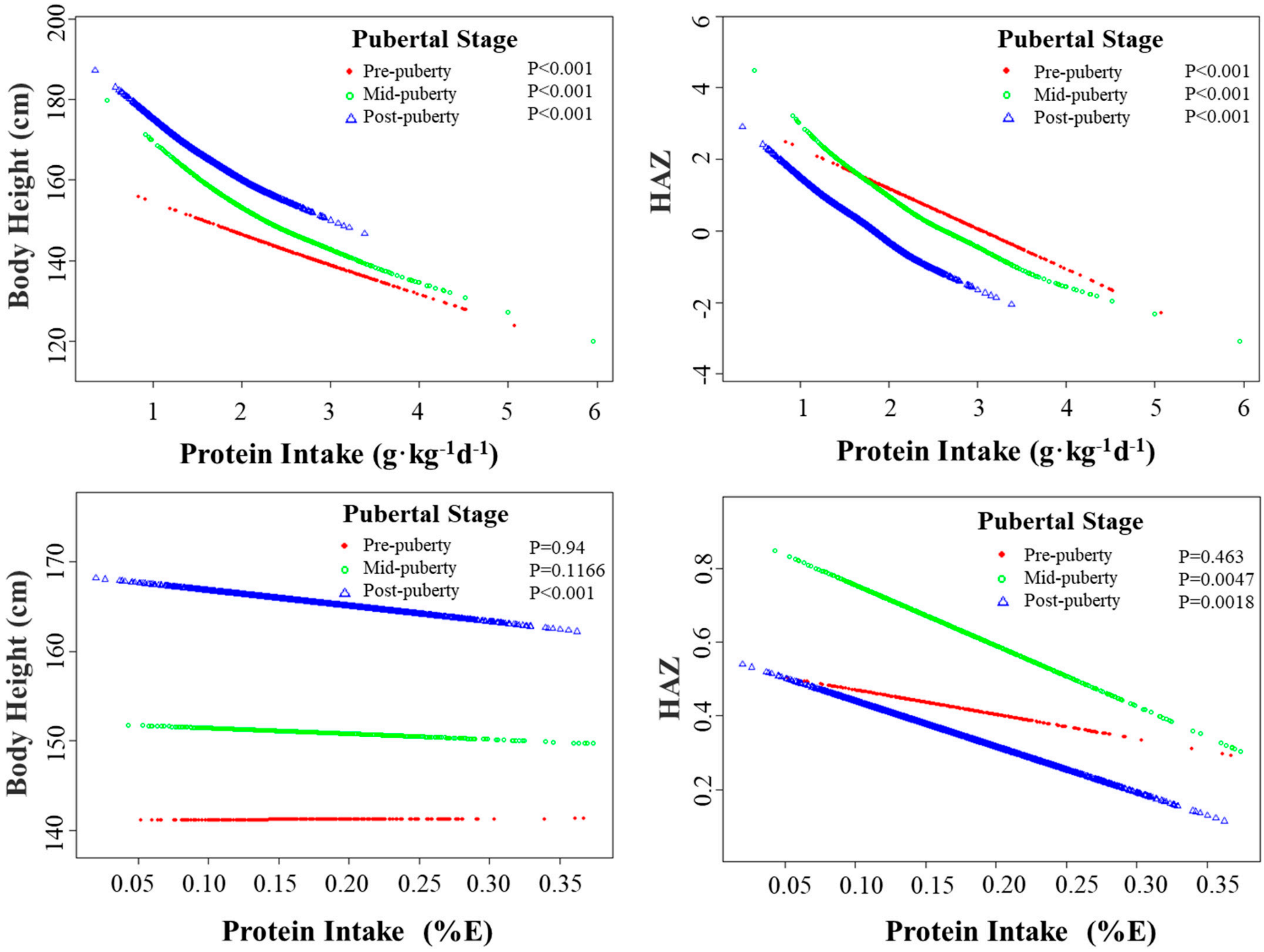

Supplemental Figure S4. The pubertal stage-specific association between dietary protein intake and linear growth.

Supplemental Table S1. Association of protein intake from different sources with body height and HAZ.

Protein Source	Protein intake, $g \cdot kg^{-1} d^{-1}$				Protein intake, %E			
	Body height		HAZ		Body height		HAZ	
	**β(95%CI)**	***P***	**β(95%CI)**	***P***	**β(95%CI)**	***P***	**β(95%CI)**	***P***
**Animal protein**								
Crude model	-6.74 (-7.09, -6.38)	<0.001	-0.14 (-0.18, -0.11)	<0.001	-1.80 (-2.22, -1.39)	<0.001	-0.06 (-0.09, -0.02)	0.001
Model I [a]	-6.48 (-6.88, -6.08)	<0.001	-0.92 (-0.98, -0.87)	<0.001	-0.81 (-1.13, -0.50)	<0.001	-0.08 (-0.12, -0.03)	0.001
Model II [a]	-6.49 (-6.90, -6.08)	<0.001	-0.93 (-0.99, -0.87)	<0.001	-0.81 (-1.12, -0.49)	<0.001	-0.08 (-0.13, -0.03)	<0.001
**Plant protein**								
Crude model	-8.31 (-8.62, -8.00)	<0.001	-0.20 (-0.24, -0.17)	<0.001	-1.83 (-2.25, -1.41)	<0.001	-0.10 (-0.13, -0.06)	<0.001
Model I [b]	-5.72 (-6.05, -5.39)	<0.001	-0.81 (-0.85, -0.76)	<0.001	-0.77 (-1.04, -0.50)	<0.001	-0.08 (-0.12, -0.04)	<0.001
Model II [b]	-5.72 (-6.06, -5.39)	<0.001	-0.82 (-0.87, -0.77)	<0.001	-0.77 (-1.04, -0.50)	<0.001	-0.08 (-0.12, -0.04)	<0.001

Abbreviations: HAZ, height-for-age z score.

A generalized linear model was used. Protein intake was set to 1 SD increase of grams per kilogram weight per day ($g \cdot kg^{-1} \cdot d^{-1}$) or percentage of total energy intake (%E). Crude model, not adjusted for any variables; Model I, adjusted for gender, ethnicity, age (as a continuous variable), pubertal stages, premature, birth length, maternal age (as a continuous variable), parental education levels (as a continuous variable), parental height (as a continuous variable), household income levels, smoking status, drinking status, moderate to vigorous physical activity (MVPA), central obesity, nighttime sleep duration (as a continuous variable), dietary fat intake (as a continuous variable), and dietary carbohydrate intake (as a continuous variable); Model II, additional adjustment for serum calcium, zinc, vitamin D_3, and vitamin A levels as continuous variables.

[a] The models were additionally adjusted for plant protein intake.

[b] The models were additionally adjusted for animal protein intake.

Supplemental Table S2. Stratified analyses of potential modification effect for the association of dietary protein intake with linear growth.

Stratification factors	N	Body height			HAZ		
		β(95%CI)	*P* value	*P* for interaction	β(95%CI)	P value	*P* for interaction
**Gender**				<0.001			0.7741
Boy	1837	-10.77 (-11.35, -10.19)	<0.001		-1.23 (-1.31, -1.14)	<0.001	
Girl	1462	-6.92 (-7.52, -6.33)	<0.001		-1.21 (-1.30, -1.12)	<0.001	
**Pubertal stage**				<0.001			<0.001
Pre-puberty	343	-6.38 (-7.17, -5.59)	<0.001		-0.98 (-1.09, -0.86)	<0.001	
Mid-puberty	887	-9.73 (-10.35, -9.10)	<0.001		-1.30 (-1.40, -1.21)	<0.001	
Post-puberty	2069	-11.31 (-12.12, -10.50)	<0.001		-1.41 (-1.53, -1.29)	<0.001	

Abbreviations: HAZ, height-for-age z score.

A generalized linear model was used. Protein intake was set to 1 SD increase of grams per kilogram weight per day ($g \cdot kg^{-1} \cdot d^{-1}$). Adjusted for gender, ethnicity, age (as a continuous variable), pubertal stages, premature, birth length, maternal age (as a continuous variable), parental education levels (as a continuous variable), parental height (as a continuous variable), household income levels, smoking status, drinking status, moderate to vigorous physical activity (MVPA), central obesity, length of nighttime sleep (as a continuous variable), dietary fat intake (as a continuous variable), and dietary carbohydrate intake (as a continuous variable), and serum calcium, zinc, vitamin D_3, and vitamin A levels as continuous variables. Analyses that were stratified by gender (boy or girl), pubertal stage (pre-puberty, mid-puberty, or post-puberty), and each of the other groups were adjusted for all covariates except itself.
